# The Ligands for Human IgG and Their Effector Functions

**DOI:** 10.3390/antib8020030

**Published:** 2019-04-25

**Authors:** Steven W. de Taeye, Theo Rispens, Gestur Vidarsson

**Affiliations:** 1 Sanquin Research, Dept Immunopathology and Landsteiner Laboratory, Amsterdam UMC, University of Amsterdam, 1066 CX Amsterdam, The Netherlands; t.rispens@sanquin.nl; 2 Sanquin Research, Dept Experimental Immunohematology and Landsteiner Laboratory, Amsterdam UMC, University of Amsterdam, 1066 CX Amsterdam, The Netherlands; g.vidarsson@sanquin.nl

**Keywords:** Antibodies, IgG, Fc effector molecules, allotypes, glycosylation

## Abstract

Activation of the humoral immune system is initiated when antibodies recognize an antigen and trigger effector functions through the interaction with Fc engaging molecules. The most abundant immunoglobulin isotype in serum is Immunoglobulin G (IgG), which is involved in many humoral immune responses, strongly interacting with effector molecules. The IgG subclass, allotype, and glycosylation pattern, among other factors, determine the interaction strength of the IgG-Fc domain with these Fc engaging molecules, and thereby the potential strength of their effector potential. The molecules responsible for the effector phase include the classical IgG-Fc receptors (FcγR), the neonatal Fc-receptor (FcRn), the Tripartite motif-containing protein 21 (TRIM21), the first component of the classical complement cascade (C1), and possibly, the Fc-receptor-like receptors (FcRL4/5). Here we provide an overview of the interactions of IgG with effector molecules and discuss how natural variation on the antibody and effector molecule side shapes the biological activities of antibodies. The increasing knowledge on the Fc-mediated effector functions of antibodies drives the development of better therapeutic antibodies for cancer immunotherapy or treatment of autoimmune diseases.

## 1. Introduction

The human adaptive humoral immune system is dependent on antigen recognition via the B cell receptor on naïve B cells, which initiates B cell maturation and eventually production of antibodies by plasmablasts and plasma cells. IgM is the initial antibody class that is made when naïve B cells are activated and can be found as a membrane-bound B cell receptor (BCR) on naïve B cells together with IgD. Like all immunoglobulins, the basic secreted unit is a dimer of two identical heavy chains, each coupled to identical light chains. For IgM, five such units associate together with a Joining (J) chain forming a pentamer, which is a strong activator of the classical complement pathway [1]. Class switching from the initial IgM isotype allows the humoral immune system to engage with each antigen in a specific manner, with unique effector mechanisms being imprinted by each class (IgM, IgG, IgA, IgE, and IgD). Additionally, IgA and IgG are further subdivided in two and four subclasses, respectively (IgA1-2 and IgG1-4). Although the IgA subclasses seem to have similar if not identical effector functions, the abundance at different locations (serum/mucosa) is very different. The effector functions of IgG subclasses are very different and will be a major topic of this review.

During the onset of initial class switching in a given B cell any class switching event is theoretically possible from IgM to any other isotype. However, further sequential class switching events are dependent on the order of the Ig heavy chain constant genes on chromosome 14 (IgM, IgD, IgG3, IgG1, IgA1, IgG2, IgG4, IgE, and IgA2) [2]. This is because of genetic excisions of constant regions, e.g., the exons encoding for IgM, IgD, and IgG3 constant regions are deleted after a class switch event from IgM to IgG1, preventing descendants of the proliferating B cell from generating IgG3. These class switching events of naïve B cells in the germinal center during clonal expansion are not completely random, but are regulated through signals received from T-helper cells and antigen presenting cells (APC). Cytokines produced by T-helper cells and signaling via toll-like receptors (TLR) on B cells initiate class switching of antigen specific B cells via activation-induced deaminase (AID) activity [3]. 

All the immunoglobulin isotypes have their own biodistribution, function and are often elicited upon specific triggers. IgD, for example, may be found in a secreted form, mostly in the tonsils, but its function remains enigmatic [4,5]. IgE is known to interact with mast cells to trigger the release of histamine mostly through the high affinity IgE-Fc Receptor I (FcεRI), but it also interacts with the atypical FcεRII (CD23), a c-type lectin. IgA has differential function depending on whether it is secretory IgA (SIgA) or serum IgA. SIgA is a dimeric form containing the J-chain (also found in IgM) that is associated with the extracellular domain of the polymeric Ig-Receptor (pIgR), which cleaves off after the transcytosis of dimeric IgA by the pIgR on epithelial cell of the mucosa [6]. Only serum IgA, which is monomeric and not associated with the J-chain, can bind and activate the myeloid IgA-receptor FcαRI efficiently and trigger a strong cellular response [7,8,9]. These isotypes—IgA, IgE, and IgD—generally do not activate complement, and therefore rely on other mechanisms to carry out their function [5,10,11]. Thus detailed discussion of these isotypes is beyond the scope of this chapter where we will focus on the biology of IgG subclasses.

## 2. Immunoglobulin G (IgG)

**Figure 1:**
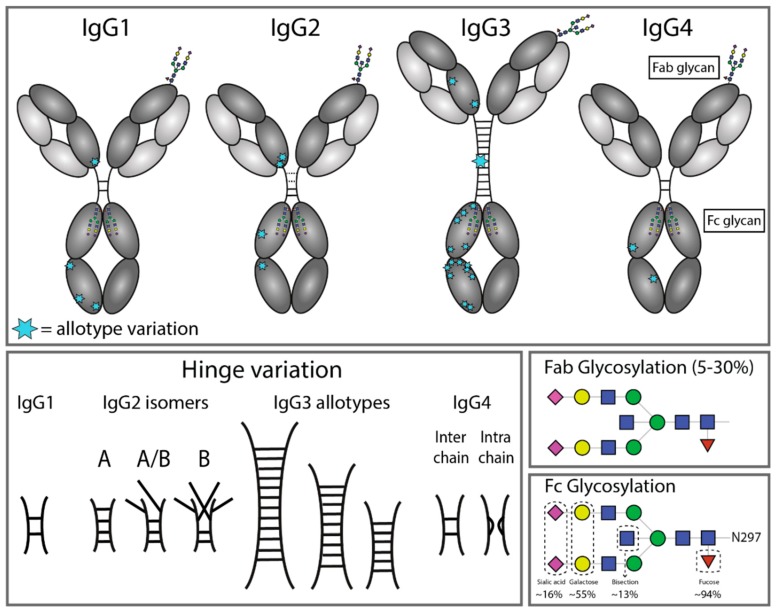
Structural variation of immunoglobulin G subclasses. A structural representation of the immunoglobulin G (IgG) subclasses and the variation within these subclasses, including allotype variation, hinge variation, and glycosylation. The variation originating from allotypic polymorphisms in the immunoglobulin heavy gamma (IGHG) constant domain is indicated by blue stars. Except for the star representing the variation in hinge length between IgG3 allotypes, each blue star indicates amino acid variation at one particular residue in the constant domain. Fab glycosylation is indicated and is present in 5–30% of antibodies in serum, depending on subclass and antigen specificity. The glycoform of the N297 Fc glycan is highly variable, for which the frequency of each glycan moiety on IgG antibodies in human serum is indicated.

In the majority of humoral antibody responses, whether it is the protection against viral or cellular pathogens, IgG-mediated effector functions are involved. This includes humoral responses in allo- or autoimmune diseases. IgG1 is the most abundant antibody in human sera, followed by IgG2, IgG3, and IgG4 respectively [12]. Although the IgG subclasses are more than 90% identical on the amino acid level, each IgG subclass has a unique profile with respect to structure, antigen binding, immune complex formation, complement activation, triggering of FcγR, half-life, and placental transport [12] (Figure 1). IgG1, IgG3, and to some extent, IgG4 are generally formed against protein antigens, while IgG2 is the major subclass formed against repetitive T cell-independent polysaccharide structures found on encapsulated bacteria [13]. IgG3 is often the first subclass to form, which is followed by IgG1 responses that later dominate. The development of IgG4 responses is often the outcome of repeated or prolonged antigen exposure, although class switching from IgM expressing naïve B cells to IgG4 is possible [14]. The unusually weak CH3–CH3 interactions in the Fc domain of IgG4 and the redox sensitive disulfide bonds in the hinge of IgG4 facilitate exchange of two half-molecules (each consisting of one heavy and one light chain from a single IgG4 molecule), which enables the formation of bispecific IgG4 molecules [14,15,16]. For IgG2, two isoforms (IgG2 A/B) exist as a result of different disulfide bonding in the Fab and hinge domain, which determines the rigidity of the Fab domains when engaging antigen [17]. Functionally, IgG1 and IgG3 are strong inducers of Fc-mediated effector mechanisms, such as antibody-dependent cellular cytotoxicity (ADCC), complement dependent cytotoxicity (CDC), and antibody-dependent cellular phagocytosis (ADCP). IgG2 and IgG4, on the other hand, generally induce more subtle responses, although IgG2 has been shown to be quite capable of inducing good complement and Fc-receptor-mediated responses against epitopes of high-density such as polysaccharides [18,19]. This capacity of IgG2 may be related also to the peculiar rigidity of the hinge, shown to result in super agonistic antibodies and triggering strong signaling when targeting immune costimulatory receptors such as CD40 [20]. Below, the interaction of different ligands with human IgG subclasses is discussed as well as the effector functions triggered via these interactions.

## 3. IgG-Fc-Engaging Effector Molecules

**Figure 2:**
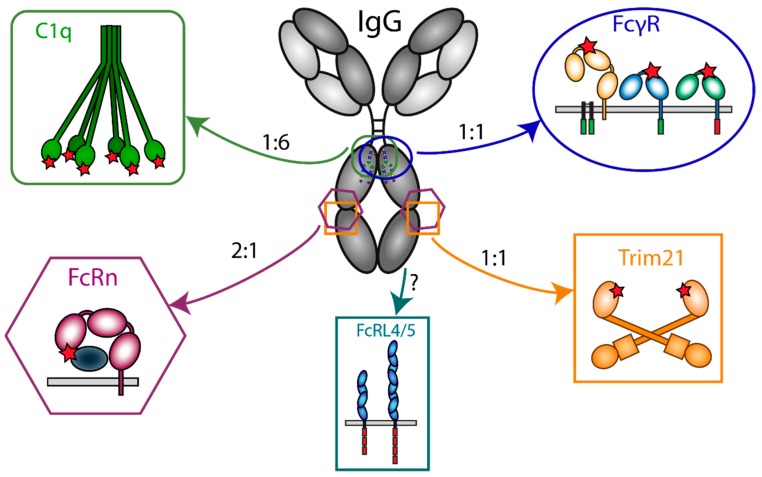
Interaction of IgG with Fc effector molecules. Schematic representation of IgG- and all Fc-engaging molecules (Complement component (C1q), Fc gamma receptors (FcγR), the Neonatal Fc receptor (FcRn), Tripartite motif 21 (Trim21), and Fc receptor-like (FcRL) molecules 4 and 5) through which antibodies exert their biological activity. For each ligand the binding site on IgG and the stoichiometry of the interaction with IgG is indicated. The red stars represent the binding site of IgG on the Fc effector molecules.

The Fc domain of antibodies is the target for many proteins, including receptors on myeloid cells and thereby serves as a ligand for adaptor molecules (Figure 2). Many biological activities of antibodies are dependent on the interaction with these effector molecules, comprising Fc gamma receptors (FcγR) [21], two members of the Fc receptor-Like (FcRL) family (FcRL4 and FcRL5) [22], complement components (C1q) [23], neonatal Fc receptor (FcRn) [24], and Tripartite motif-containing protein 21 (TRIM21) [25].

### 3.1. Fc-Receptors

Human myeloid, NK, and some lymphoid cells express Fc gamma receptors (FcγR), which sense antibody-opsonized particles and exert their specific effector mechanisms upon recognition and clustering of the Fc receptors [26]. Based on monovalent IgG:FcγR binding studies, FcγR were classified into high-affinity (FcγRIa) and low-affinity (FcγRIIa, IIb, IIc, IIIa, and IIIb) receptors [27]. This classification is somewhat of an oversimplification, as, for example, the affinity of IgG1 to FcγRIIIa can approach that of FcγRI depending of fucosylation in the IgG1-Fc (see below). These affinities also are not always indicative of their differential functionalities as it is the cross-linking of these Fc-receptors, brought about by engagement with immune complexes or opsonized pathogens with multiple IgG molecules, which enables the initiation of signaling. [28,29]. 

Most FcγR associate with an intracellular immunoreceptor tyrosine-based activation motif (ITAM), which is either directly found in the cytoplasmic domain (FcγRIIa and FcγRIIc-ORF) or through the associated FcRγ-chain (FcγRIa and FcγRIIIa). The exceptions are FcγRIIIb, which is GPI-linked, and FcγRIIb, which has an immunoreceptor tyrosine-based inhibition motif (ITIM). The latter is therefore the only receptor with inhibitory activity, and the only Fc receptor expressed on B cells [30]. The ratio of activating and inhibitory (A:I) FcγR expression on immune cells is thought to determine the antibody threshold necessary to activate the effector cell and induce ADCC or ADCP [31]. The FcγR expression pattern is highly variable between different immune cells. NK cells, for example, only express the low affinity FcγRIIIa receptor, while macrophages and monocytes express multiple receptors (FcγRIa, IIa, IIb, and IIIa) [26]. Depending on the Fc receptor a range of different effector functions can be triggered via the interaction with IgG, for example, binding of FcγRIIIa to IgG opsonized viruses or infected cells facilitates cross-linking of FcγRIIIa, which initiates ADCC of the target cell. 

Fc gamma receptors bind the Fc domain of IgG in a 1:1 stoichiometry via interactions with the lower hinge (residues 234–238), the CH2 domain (residues 265, 297, 298, 299, and 329), and the N297 Fc glycan [32]. All FcγRs bind to IgG via their second extracellular domain, which shows great structural homology between the Fc receptors (root mean square deviation of atomic positions <1.0 Å). While all low-affinity receptors have two extracellular domains, the high-affinity receptor FcγRIa consists of three domains. The interaction of antibodies with various FcγRs is influenced by the IgG subclass. IgG1 and IgG3 bind efficiently to all Fc gamma receptors, contributing to their overall strong effector function profile [28]. The affinity of IgG4 for FcγRIa is two-fold lower compared to IgG1 and IgG3 (K_A_ 3.4 × 10^7^ M^−1^) [28]. IgG4 binds very weakly to the other FcγRs, which only leads to activation in situations where multivalency/avidity are involved [28]. IgG2 lacks a leucine at position 235 in the low hinge of Fc that is critical for binding to the high affinity receptor FcγRIa. This may therefore be an important reason why IgG2 does not bind FcγRIa [32]. Binding of IgG2 to FcγRIIa and FcγRIIIa is of low affinity (K_A_ of 10e^6^ and 10e^5^ M^−1^, respectively), which is functionally relevant in the recognition of IgG2 immune complexes particularly through FcγRIIa [28,33]. Of note, IgG2, which is almost exclusively found as fucosylated species in humans, does show elevated binding to FcγRIIIa when afucosylated [33]. However, despite measurable binding to FcγRIIIa, afucosylated IgG2 only showed a slight albeit not significant increase in ADCC by NK cells (<5% killing) using IgG2-anti-Rhesus D opsonized RBC [33].

FcγR are highly polymorphic, thus their exact composition differs from person to person, and the ethnic makeup is also variable [21]. Not all FcγR-allotypic variation seems to have functional consequences, but a few polymorphisms are particularly noteworthy. Polymorphic variants of FcγRIIa (131H/R) and FcγRIIIa (158F/V) have different binding affinities to IgG. Thus, in contrast to monomeric IgG1 and IgG3, IgG2 has a particularly strong preference for FcγRIIa-131H compared to the FcγRIIa-131R variant [28,33]. The polymorphic variant FcγRIIIa 158V binds IgG1 and IgG3 with a 5-fold stronger affinity compared to FcγRIIIa 158F [33]. One allotypic variation results in the lack of expression of the FCGR2C gene, which is a pseudogene in most individuals and most ethnic groups [34]. However, in ~7–15% of individuals of European origin, FcγRIIc (FCGR2C-ORF) is expressed on some immune cells, including NK cells and perhaps B cells [35,36,37]. FcγRIIc expression depends on the presence of a single nucleotide polymorphism in exon 3 of FCGR2C, which normally encodes a stop codon (FCGR2C-Stop) [38]. Curiously, in some individuals with a FCGRIIC-STOP allele, FcγRIIb expression has been found on NK cells, which is normally absent for this cell type. Although the reason is unknown, this phenotype is accompanied by a genetic deletion of the FCGR2C and FCGR3B genes adjacent to FCGR2B on one chromosome, perhaps because this results in a net replacement of the FCGR2B promotor with the promotor of FCGR2C [36].

In line with the differences in interaction with the IgG subclasses, FcγR polymorphisms were found to correlate with IgG-dependent diseases, such as in allo- and autoimmunity [39,40,41], and with outcome of treatment in therapeutic antibody regimens that trigger FcγR for its therapeutic effect [21]. RA patients receiving rituximab treatment targeting CD20 on B cells generally respond better when bearing the higher-affinity FcγRIIIa 158V variant [42,43]. This advantage of expressing polymorphic variant FcγRIIIa 158V was less conclusive in other patient groups receiving rituximab, for example patients with non-Hodgkin’s lymphoma [44,45]. In addition to single nucleotide polymorphisms in the FCGR-gene locus that alter interaction with the IgG Fc domain, copy number variation (CNV) also influences FcγR expression in a gene dosage fashion [36,46,47]. 

### 3.2. DC-SIGN and CD23

In addition to the type I Fc receptors that we discussed above, a second class of Fc receptors (type II) has been described to bind to the CH2:CH3 interface of sialylated IgG Fc [48,49]. These are the C-type lectin homologs DC-SIGN and CD23, of which the latter is the low-affinity receptor for IgE, which has been proposed to embody another group of Fc engaging molecules. The sialylated Fc fraction of IVIG was proposed to be responsible for the anti-inflammatory mechanism of IVIG, through binding of these type II Fc receptors. Structural studies of sialylated Fc revealed that the overall conformation is comparable to nonsialylated Fc, implying that the CH2:CH3 interface is not altered when the Fc-glycan is sialylated [50]. In fact, we and others found no detectable binding of human DC-SIGN and CD23 to human IgG Fc by FACS or SPR [51,52]. There is thus discrepancy between those reporting a biological effect of IgG through these receptors [48,49,53,54] and those finding no effect [50,51,52,55]. However, the overall consensus seems now to indicate that DC-SIGN and CD23 are not bona fide receptors for human IgG. 

### 3.3. FcRn

The neonatal Fc receptor (FcRn) belongs to the family of MHC-related proteins and enables transcytosis of IgG over the maternal placenta. In addition, it mediates the long half-life of IgG. FcRn is rather ubiquitously expressed, including endothelial cells aligning our vessel walls, with a particularly high expression on myeloid cells [56]. Binding of IgG by FcRn occurs exclusively at low pH in endosomes (pH < 6.5) after IgG is engulfed into endosomes via pinocytosis. Thereafter, FcRn shuttles IgG back to the cell membrane, where IgG dissociates from FcRn after fusion with the surface at a pH of 7.4 in the extracellular fluid. This pH-dependent interaction results from the binding of FcRn with two histidines at the CH2:CH3 interface (H310 and H435), which become protonated at low pH (pKa around 7.4) [57,58]. This property of the IgG–FcRn interaction is critical to allow for pH-dependent FcRn-mediated shuttling of IgG. This increases the half-life of IgG1, IgG2, and IgG4 to ~21 days. Most IgG3 allotypes lack the histidine at position 435, and instead have an arginine (pKa ~12.5) at this position, which is always positively charged, independent of the physiological pH found in endosomes or extracellular medium. As a consequence, it is recycled to a much lesser extent than the other subclasses, and therefore has a much shorter half-life of ~7 days [59] and has less transport across the placenta [60,61]. However, some IgG3 allotypes express the histidine at position 435 (homologous to the other subclasses) and therefore have an increased half-life and transcytosis [59,60,61]. Interestingly, charge variations in the Fab domains of IgG have recently also been found to affect the half-life of IgG, suggesting that domains outside the binding site affect pinocytosis levels and/or in vivo FcRn binding kinetics [62,63]. In the proposed model, this effect may be due to the positively charged Fab domains affecting the interaction with the negatively charged cell membranes through the primary interaction site in the IgG-Fc. 

### 3.4. TRIM21

Tripartite motif 21 (TRIM21) is an intracellular cytosolic IgG receptor which recognizes the CH2:CH3 interface, a binding site partly overlapping with that of FcRn. TRIM21 is a dimeric molecule, containing two PRYSPRY binding domains, which bind both CH2:CH3 elbow domains in a pincer-like interaction [25]. One PRYSPRY domain binds IgG with a reported affinity of 130nM [64,65]. However, the affinity of TRIM21 for IgG increases ~250-fold when dimeric TRIM21 binds both IgG heavy chain CH2:CH3 domains simultaneously (reported affinity being 0.6nM) [66]. A histidine at position 433 in the Fc domain of IgG is critical for binding of TRIM21 to the IgG Fc domain [67]. Entry of an IgG-opsonized non-enveloped virus or intracellular bacteria is recognized by TRIM21 and binding of TRIM21 triggers polyubiquitinylation of the opsonized particles and proteasomal degradation, as well as transcriptional activation of several immune regulator genes through the NF-κB, AP-1, and several IRF genes [25,67,68]. This process is described as antibody-dependent intracellular neutralization (ADIN), and extends the effector functions of antibodies to the intracellular compartment of cells. Whether strong immune signaling is initiated via activation of transcription factors, depends on the affinity of the antibody for the antigen/pathogen [67]. Viral particles opsonized with high affinity antibodies trigger transcriptional activation of immune regulators and production of cytokines [67]. So far, the evidence seem to suggest that early immune responses against cytosolic pathogens, probably non-enveloped viruses in particular, can be counteracted by the early immune response as TRIM21 also recognizes IgM and IgA [25]. Recognition of cytosolic immunoglobulins by TRIM21 may also function as a back-up mechanism in secondary infection for regular extracellular antibody mediated effector mechanisms when normal cellular compartmentalization has been compromised. 

### 3.5. FcRL

Fc receptor-like (FcRL) molecules are part of the immunoglobulin superfamily (IgSF). In the human context, six transmembrane receptors—FcRL1–6—are described, consisting of three to nine extracellular Ig-like domains and several ITIM and/or ITAM signaling molecules intracellularly. These receptors are expressed on B cells, and the expression pattern of FcRLs varies during the different stages of B cell development [69]. In addition, FcRL3 and FcRL6 are also expressed on certain NK and T cell subsets [70,71,72]. The ligands of FcRL molecules and the processes in which signaling via FcRL is involved are not completely understood. However, FcRL4 (four extracellular immunoglobulin domains) and FcRL5 (eight extracellular immunoglobulins) were found to bind immunoglobulins with low affinity. FcRL4 specifically binds IgA, IgG3, and IgG4, while FcRL5 is able to bind all IgG subclasses [73,74]. The exact binding epitope of FcRL5 on IgG was not determined. However a complex binding interaction was proposed, in which both the Fc and Fab domains of IgG are involved in the interaction with FcRL5 [74]. Each receptor express several classical and atypical ITIM-motifs, as well as a potential ITAM-motif [75]. Both receptors are found on B cells suggesting a role in regulation of humoral immune responses [76]. One recent study suggested that FcRL5 can induce either inhibiting or activating signals to B cell receptor signaling pathways, depending on coligation with complement receptor 2 (CD21) [77]. This seems to suggest that complement C3 deposition may convert an inherent inhibitory signal to an activating signal and positively stimulate the humoral immune response. However, the exact mechanism and significance for human immune responses has not been elucidated.

### 3.6. Complement (C1q)

Binding of C1q to monomeric IgG requires clustering of IgG to establish a multivalent interaction platform. This platform resembles that of an IgM molecule, which consists mostly of a pentameric structure, but can also form a hexameric structure, in the absence of a Joining (J) chain, and binds C1q in a 1:1 stoichiometry when bound to antigen [1,78]. Recently, it has been determined that IgG bound to a membrane structure is able to assume a hexameric configuration in complex with C1 thereby initiating complement activation. The structural characterization of an IgG1 hexamer in complex with C1q unraveled some important features of this interaction [79]. Two major interaction sites determine binding of the globular head domain of C1q to IgG Fc. The BC loop (residues 266–272) and DE loop (294–300) of one CH2 domain are part of the first site and the FG loop (325–331) of the other CH2 domain is the second site [79]. Hexamer formation does not take place in solution to a measurable degree because the interactions between the individual IgG Fc tails are very weak. However, upon binding a cellular surface, multiple antibodies are confined into a limited space, favoring Fc–Fc interactions between these IgG molecules, which is expected to be even more favorable in the presence of C1q. IgG hexamer formation is impaired when C-terminal lysines on IgG enforce charge repulsion between IgG molecules. The C-terminal clipping of these lysines residues by plasma carboxypeptidase is necessary to facilitate efficient hexamer formation and complement activation by IgG [80]. IgG is produced in a proform and C1q binding is also modulated by the glycoform of the N297 Fc glycan, with increasing galactosylation stimulating better C1q binding and downstream activation (C3, C4 deposition, and CDC) [81,82,83]. Multivalent binding of IgG Fc condenses the C1q arms, which drives the rearrangements of the C1r2s2 proteases allowing catalytic activity of these complement components [79]. In this particular structure an IgG1 hexamer was studied, although a structurally similar principle should apply to IgG3 despite its significantly longer hinge. Binding and activation of C1q is in general believed to be more efficient with IgG3 compared to IgG1, although in certain cases CDC with IgG1 sometimes seems to outperform IgG3 [84,85,86]. The molecular reasons for this remain unresolved.

## 4. IgG Allotypes

Similar to FcγR, allotypic variations also exist for antibody heavy chains, especially for IgG. These polymorphisms form another layer of variation that may influence functional and structural features on top of what we know for the IgG subclasses [12]. Multiple studies found IgG polymorphisms to be associated with susceptibility to infectious diseases and autoimmune diseases, suggesting that IgG allotypes affect the humoral antibody response [87,88,89,90,91,92]. However, the structural or functional characteristics of IgG allotypes underlying these associations have not yet been elucidated. 

The IgG allotypic background of an individual correlates with the individual IgG—subclass plasma levels [93,94]. A possible explanation may be the result of the formation of noncoding transcripts or RNA transcripts with unfavorable codon composition that impedes transcription and/or translation. In addition, IgG polymorphisms may also be associated with altered class switching efficiency, through variations within the noncoding switch regions, which would subsequently affect serum concentrations [93,95,96]. In a recent study, Shattock and colleagues showed that IgG1 allotypic variants were associated with the subclass distribution (IgG1/IgG2) of an HIV-specific antibody response, illustrating the association of IgG polymorphisms with the tendency for particular IgG subclass switching [97].

In addition to the association of IgG allotypes with antibody expression and IgG class switching in B cells, the Fc-mediated effector functions may be different between IgG allotypes. Previous studies already identified IgG3 allotypes with less stable CH3–CH3 interactions and an IgG4 allotype lacking the capacity to exchange half-molecules [16,59,98,99]. Furthermore, a particular IgG3 isoallotype (IMGT: IGHG3-17, -18, and -19) expressing a histidine at position 435 in the CH3 domain was found to improve pH-dependent binding to the neonatal Fc receptor (FcRn) and therefore showed an half-life that resembled that of IgG1 antibodies [59]. Also, infants of mothers carrying this IgG3 polymorphic variant were found to have an increased protection against malaria, since the malaria specific IgG3 antibodies crossed the placental membrane more efficiently as a result of increased binding to FcRn [60,61]. 

Future studies will shed light on the effector functions of the various IgG allotypes and the potential implications in susceptibility to infectious diseases or translation to antibody-based therapeutics. Interestingly, reactivity of monoclonal or polyclonal anti-IgG antibodies with all IgG allotypes indicated that some monoclonal antibodies, either subclass- or isotype-specific do not recognize all allotypic variants, a phenomenon described as ‘serological blind spots’ [100,101]. In addition, subclass-specific polyclonal anti-IgG were found to react with isoallotypic variants of another subclass, which could lead to misinterpretation of IgG subclass responses and can be of great importance, not only for scientific interpretations of immune responses, but also for critical diagnostic conclusion that leads to life-and-death decisions for patients [100].

## 5. IgG Glycosylation

Both heavy chains of the IgG express an N297-linked glycan in the CH2-domain in the Fc regions, which have a role in stability of the Fc domain and in the interaction with FcγR and possibly C1q [102]. During antibody production in the plasmablasts, the presence and activity of glycan-processing enzymes determines the composition of Fc-glycans, which results in a heterogeneous glycosylation pattern on antibodies. 

Fucosylated complex glycans, with low-to-intermediate levels of galactosylation and low sialylation are most commonly found in serum IgG-Fc. Although not commonly found in plasma, afucosylated glycan-species of the N297 glycan (found in ~6% of plasma IgG) were previously found to increase the binding strength of IgG-Fc to FcγRIIIa [81,103,104]. This leads also to enhanced ADCC and has already been exploited in some therapeutic antibodies to improve their effector functions [105]. The molecular reason for the enhanced binding to the FcγRIII-family of receptors has been enigmatic and has still not fully been uncovered, although it is known to depend on a glycan found at position 162 only in human FcγRIII (a and b) (and conserved in other species, e.g., mice have FγRIV also with N162) [106]. Recent work based on structural modeling provided evidence that the number of conformations sampled by the N162-glycan is reduced by the presence of the fucose in the IgG Fc glycan [107,108]. This work suggests that the fucose moiety on the Fc glycan may be affecting the N162-glycan mobility which partially inhibits FcγRIIIa/b to engage in binding. Thus, without the fucose, the IgG-glycan can more effectively make room for effective FcγRIIIa/b binding. These notions are also supported by the fact that removing the N162 glycan of FcγRIIIa also increases the affinity to IgG which is no longer affected by the fucosylation status of the IgG [106,107,108,109]. 

In recent years glycoengineering has been utilized to develop next generation afucosylated therapeutic antibodies with enhanced ADCC activity. For example, an afucosylated anti-CD20 antibody has been approved to treat patients with B cell lymphomas [105]. In addition to fucosylation, galactosylation and sialylation of the N297 glycan were also described to modulate IgG effector functions [33,81,82,83,110]. Whereas galactosylation of IgG seems to increase complement activation, sialylation has both been reported to decrease CDC activity of rituximab-IgG anti-CD20, but in other cases to increase RBC lysis of anti-D IgG1 [81,83]. As the architecture of the immune complex formed by the anti-CD20 and anti-D may differ, this may offer an explanation for the differential outcome [111]. Both studies were carried out using normal human serum as complement source (albeit at different concentrations 5% and 10%). This may also offer a potential explanation as difference in serum composition (e.g., endogenous IgG glycosylation status and immune complex formation) may affect C1q binding to the intended target as we recently suggested [112]. 

Increased complement activation by IgG Fc galactosylation may also seem at odds with findings in several autoimmune diseases, where a low degree of galactosylation of total IgG-antibodies was found to be associated with disease progression [112]. Recently, we put forward a model that may explain how this is possible, taking into account the relative difference between the glycosylation of the pathogenic antibodies (in most cases not determined) and total IgG that may affect the threshold of immune activation [112]. This is because the bulk of aspecific IgG will always account for significant occupation of both FcγR but also partly C1q. 

In addition to the conserved N-linked glycan in the Fc-domain [113], potential N-glycosylation sites are also present in the variable domain of antibodies. These Fab glycans have been described to modulate antibody stability, but also antigen binding directly [114,115]. As such, we recently postulated these sites to be a fundamental enhancement to the generation of antibody diversification—on top of VDJ recombination and somatic hypermutation leading to amino acid changes [114]. Fab glycosylation was found to be isotype and subclass-specific and associated with several autoimmune diseases including rheumatoid arthritis (RA) and primary Sjögren’s syndrome [116,117,118]. Anticitrullinated protein antibodies (ACPA) are formed in the majority of RA patients and ACPA positive patients have an increased risk for rapid disease progression [119]. ACPA more frequently harbor Fab glycans compared to total serum IgG, suggesting that B cells producing auto-antibodies with Fab glycans are positively selected during affinity maturation [120]. In addition, the dominant Ig-producing cells in parotid glands of Primary Sjögren’s syndrome were found to frequently express Fab glycans in the variable domain of the heavy chain [121]. In addition, it is possible that the existence of Fab-glycans may also affect regulation and affect the threshold for B cell activation through co-cross-linking of lectins in either cis or trans [122]. All in all, it is clear that both B cell biology, the humoral repertoire composition and the effector phase of antibodies is regulated through both Fab- and Fc-glycosylation.

## 6. Antibody Fc engineering

In recent years impressive progress has been made in the application of antibody-based therapeutics in various fields including B cell lymphomas, solid tumors and in autoimmune diseases. A popular strategy has been the generation of afucosylated antibody therapeutics to improve effector function of therapeutic antibodies for tumor immunotherapy. Both glycoengineering and protein engineering have rendered IgG Fc domains with enhanced binding to activating Fc receptors and reduced binding to inhibitory receptor FcγRIIb [81,123,124,125,126]. 

When the primary mode of action of a therapeutic antibody is to block the activity of a molecule, such as a proinflammatory cytokine, antibodies lacking Fc-mediated effector functions, ‘Fc dead’, may be desired to prevent activation and inflammation during treatment [126]. This can sometimes partly be achieved by generating antibodies with an IgG4 Fc domain, IgG2 Fc domain, combination of both or an N297-glycan deficient Fc domain. Even better is to engineer IgG-Fc variants that have no FcγR or C1q binding activity at all [127,128,129]. It should be cautioned that engineered antibodies may also express additional modes of action, e.g. afucosylated anti-TNF antibodies might have enhanced therapeutic potential in inflammatory bowel diseases. Although the exact mechanism is not known, it seems to require stimulation of wound healing macrophages through either TNF-anti-TNF- or membrane bound TNF-anti-TNF-complexes interacting with FcγRIIIa on CD206+ macrophages [130].

Another strategy to improve the efficacy of therapeutic antibodies is based on enhancing the half-life of antibodies by increasing the affinity for FcRn at low pH. Structure-based design of Fc fragments with improved affinity for FcRn at low pH was found to increase the half-life of therapeutic antibodies in vivo [131,132,133]. To stimulate the clearance of harmful auto-antibodies, FcRn blocking antibodies or Fc-fragments have been developed. Most of these antibodies bind with increased affinity to FcRn and in an pH independent fashion, thereby blocking the receptor for recycling of serum IgG [134,135,136]. Alternatively anti-FcRn antibodies are designed to bind to FcRn at the interaction site with IgG, blocking IgG recycling [137,138]. 

In addition to the structural details that determine the interaction of IgG with Fc engaging effector molecules, the context in which these interactions occur are similarly important and very relevant for the implication of antibody-based therapeutics. For example antigen density and mobility on the cell surface of target cells determine whether the bound antibodies can sufficiently trigger Fc gamma receptor cross-linking, which is a prerequisite for ADCC and ADCP. Furthermore the distance of the antigen from the cellular surface was found to be important in the initiation of effector mechanism by antibodies [139,140]. Antigen positioned close to the membrane allows for a stronger interaction between effector cell and target cell, which drives a more efficient ADCC or ADCP [139]. 

Beyond tweaking the interaction between IgG and Fc engaging molecules, Fc engineering has also been extended to the generation of bispecific antibodies. This has been realized by swapping one half (e.g., one pair of a heavy and a light chain) of a specific IgG antibody with another half of an IgG molecule with a different specificity. This allows the resulting molecule to bind two different antigens simultaneously and gives bispecific antibodies several advantages. This has for example enabled the application for antibody therapeutics including dual epitope targeting and recruitment of T cells to targeted malignant cells. Two examples of strategies to produce bispecific antibodies are controlled Fab arm exchange (cFAE) and the knob-into-hole (KIH) design [141,142,143]. The latter strategy is based on the coexpression of two antibodies, one with a knob (bulky amino acid) and one with a hole in the CH3:CH3 interface. Coexpression of the knob and hole heavy chains with a common light chain followed by protA affinity chromatography leads to 95% heterodimerization efficiency [141,142]. The other strategy (cFAE) is also known as the DuoBody platform [143]. For each application of a bispecific antibody, whether that is cancer immunotherapy or neutralizing infectious agents, the desired features are different. This is why many different bispecific antibodies are developed based on full IgG (bsIgG) or single chain/variable domain only (scFv) [144,145].

In conclusion, antibodies come in all shapes and sizes and interact with a variety of ligands to mediate effector functions. For potential protection or therapeutic applications, the appropriate format that fits the target is likely to be of utmost importance. This is further complicated by the presence of regulatory inhibitory molecules/receptor–ligand pairs found in the immunological synapse regulating myeloid and NK cell activities [146]. For prophylactic immunotherapies with antibodies, there other factors that are important to consider. This may be especially in the tumor microenvironment where checkpoint receptor–ligand receptor pairs, which can be anti-inflammatory, must be overcome before therapeutic antibodies can be of beneficial value. This can be achieved by applying more potent engineered antibodies and/or by applying a combination of antibodies targeting both tumors and checkpoint inhibitors. This will affect both myeloid and lymphoid regulatory cells and secretion profiles stimulating or inhibiting cytokines [147,148]. The variety in antibodies in terms of isotype, allotype, subclass, glycosylation profile, and specificity, together with the number of Fc engaging molecules expressed on immune cells through which effector functions are exerted, illustrate the complexity and plasticity of the antibody response. Elucidating the interactions of antibodies with Fc engaging molecules is of crucial importance in the development of antibody therapeutics.
